# Experimental Evaluation of IEEE 802.15.4z UWB Ranging Performance under Interference

**DOI:** 10.3390/s22041643

**Published:** 2022-02-19

**Authors:** Janis Tiemann, Johannes Friedrich, Christian Wietfeld

**Affiliations:** Communication Networks Institute (CNI), TU Dortmund University, 44227 Dortmund, Germany; johannes.friedrich@tu-dortmund.de (J.F.); christian.wietfeld@tu-dortmund.de (C.W.)

**Keywords:** ultra-wideband (UWB), two-way ranging (TWR), multi-user interference, IEEE 802.15.4a, IEEE 802.15.4z

## Abstract

The rise of precise wireless localization for industrial and consumer use is continuing to challenge a significant amount of research. Recently the new ultra-wideband standard IEEE 802.15.4z was released to increase both the robustness and security of the underlying message exchanges. Due to the lack of accessible transceivers, most of the current research on this is of theoretical nature though. This work provides the first experimental evaluation of the ranging performance in realistic environments and also assesses the robustness to different sources of interference. To evaluate the individual aspects, a set of three different experiments are conducted. One experiment with realistic movement and two additional with targeted interference. It could be shown that the cryptographic additions of the new standard can provide sufficient information to improve the reliability of the ranging results under multi-user interference significantly.

## 1. Introduction and Related Work

Due to its precise Time of Arrival (TOA) capabilities, Ultra-Wideband (UWB) technology emerged as the most promising candidate for the future of low-power wireless localization [1]. Its compactness and affordability challenged a significant amount of research to utilize the location estimation capabilities in industrial but also robotic contexts [2,3]. Depending on the use-case UWB can be used to obtain ranges through Two-Way Ranging (TWR) or through Time-Difference of Arrival (TDOA). While TWR based approaches have been typically limited by multi-user and anchor count scalability [4], research on TDOA based localization enabled scalable localization solutions such as [5,6,7,8].

**Figure 1:**
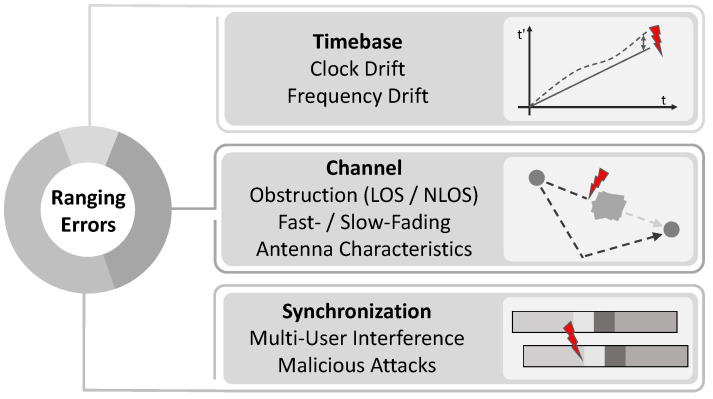
Illustration of the major ranging error sources for accurate ultra-wideband time of arrival estimation. While characteristics of the timebase are addressable through higher quality components and changes in protocol, the channel aspects are mostly immutable. Therefore, this work focuses on the parts of the IEEE 802.15.4z UWB standard that address the synchronization to ensure reliable and safe ranging.

The unique capabilities of UWB such as fine-granular time resolution enabled research on accuracy and reliability improvements such as the assessment of the channel response available at the transceiver [9,10,11,12,13]. An overview of the sources of TOA estimation errors is given in Figure 1. There are several reasons that introduce erroneous estimations. Next to problems of clock synchronization, resulting in deviating timebase, channel effects such as obstruction [14,15], fading and antenna characteristics play a major role. While these effects are mostly immutable, another aspect is multi-user interference and malicious attacks. Here, the synchronization by the preamble is affected through non-desired pulses.

In practical use these effects are observed [16,17] and some research provides measures for mitigation through Time-Division Multiple Access (TDMA) [7,18], alternative scheduling [19] or detection. While the Institute of Electrical and Electronics Engineers (IEEE) 802.15.4a-2015 standard [20] suffers from not providing methods to counter these synchronization issues, the newly introduced IEEE 802.15.4z-2020 standard [21] provides measures to reduce these effects. Next to many improvements in the allowable Physical Layer (PHY) configuration [22,23] and reduced ranging times [24], another cryptographic spreading sequence, Scrambled Time Sequence (STS) is introduced to detect and mitigate erroneous TOA estimation [25,26,27].

**Figure 2:**
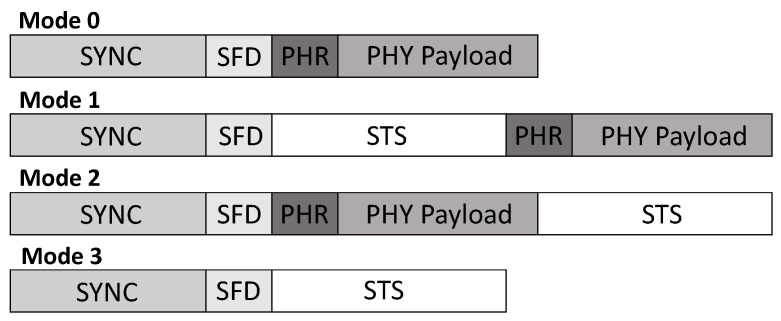
Illustration of different PHY frame formats as defined in the IEEE 802.15.4z-2020 standard. Mode 1–3 incorporate the STS. Note that different positions of the STS can enable several different functionalities, such as potential IEEE 802.15.4a backwards compatibility for Mode 2, or sending no payload at all for Mode 3.

The IEEE 802.15.4z standard defines four different PHY frame formats depicted in Figure 2. The mandatory Mode 0 uses no STS sequence, such as the previous IEEE 802.15.4a standard. Mode 1 employs an STS sequence right after the Synchronization Preamble (SYNC) and the Start of Frame Delimiter (SFD). Placing the STS before the Packet Header (PHR) and the payload allows for early discarding frames when the STS does not correlate. This format is the mandatory form when STS is to be used and payload transmission is intended. The non-mandatory Mode 2 places the STS after the payload field allowing for potential backwards compatibility to the legacy PHY frame format used in the previous IEEE 802.15.4a standard. Therefore, heterogeneous systems with legacy transceivers are possible. Another option defined by the mandatory Mode 3 is using no payload at all. This is useful when only the TOA information is of value, but payload is not necessary. Sender attribution can be conducted evaluating the STS.

Additionally to the STS, the transceiver chip also supports an STS mode with a code that is optimized for ToA performance [28]. This so called Super Deterministic Code (SDC) does not provide the security features of STS but is able to improve TOA performance. Consequently, this SDC mode is expected to perform well with untargeted unspecific interference, but is prone to targeted malicious attacks.

There is already work and proposals on how to modify the standard for scalable localization using preamble-phase TDOA [29] and on choosing the best ranging interval [24]. An analytical study on the security of UWB time-of-flight measurements has been conducted in [30], which shows the susceptibility of UWB communication to a wide range of pro-active attacks. A practical experimental proof of concept of distance reduction attacks is provided in [31], where the authors are able to interfere with one of the first widely deployed consumer UWB hardware, the *Apple U1*. Defenses against perception-layer attacks on IoT devices were investigated in [32].

However, the practical effects of the capabilities added by STS under multi-user interference have best to the authors knowledge not been extensively experimentally evaluated. Due to the wide interest in the industry [33], an experimental evaluation is even more important. Therefore, this work aims to experimentally analyze the capabilities of newly available transceivers compatible with the new IEEE 802.15.4z-2020 UWB standard under multi-user interference. With newly available *DW3000* (Qorvo Inc., Greensboro, NC, USA) family transceivers [28], a direct comparison against the performance of the widely used *DW1000* (Decawave Ltd., Dublin, Ireland) transceivers [34] is conducted in order to extract the most important differences for practical use-cases.

This work is structured as follows: We will first introduce the methodology in Section 2. Then we provide an extensive experimental evaluation in Section 3. First we will compare the ranging accuracy in a scenario covering different Line of Sight (LOS) and Non-Line of Sight (NLOS) conditions in Section 3.1. Further, in Section 3.2 we will analyze the performance and integrity under active multi-user interference in a controlled environment. This allows for relative quantification of the ranging loss and the amount of erroneous rangings under multi-user interference. Finally, in Section 4 we will conclude this work.

## 2. Experimental Methodology and Common Parameters

Since it is critical to lay out the experimental methodology and parameters for experiments conducted in this work, this section provides a brief overview of the general configuration and the detailed settings. In order to improve reproducibility, the evaluation is based on the example code provided by *Qorvo* along with their transceivers [35,36]. Additionally, we provide the raw experimental data to enable future assessment of the experimental results, see [37].

The basic common channel configurations are listed in Table 1. The experiments for the ranging pair are performed utilizing channel 5. While not exactly mandatory for IEEE 802.15.4z communication a common setting of 128 preamble symbols was chosen. Further, a Pulse Repetition Frequency (PRF) of around 62.4 MHz with a datarate *R* of around 6.8 Mbps was selected. Preamble code $c_{pr}$ 9 with a Decawave-specific preamble Preamble Acquisition Chunk (PAC) of 8 symbols was chosen. When STS is employed, the number of STS symbols $n_{sts}$ is selected to be 128 and Mode 1 is chosen for the STS placement. Please note that the SDC mode is kept at the manufacturer configuration utilizing a 64 symbol preamble, a 64 symbol STS field and a manufacturer-specific non standard SFD. The output power is left at the factory calibrated default values meeting the −41.3 dBm/MHz spectral mask for the UWB regulatory limits within the usage of the modules.

### Two-Way Ranging Configurations

The experiments in this work are evaluated using the two most basic ranging methods. Ultra-Wideband enables the precise measurement of the TOA of individual frames. Therefore, the Time of Flight (TOF) can be estimated with the knowledge of the underlying transmission and reception times. The most basic variant is Single-sided (SS) TWR. Here, the ranging procedure is initiated by the *Initiator* through the transmission of a *Poll* frame. The *Initiator* is noting the time of transmission $t_{p:tx}$. Once the other node in the ranging pair called *Responder* is receiving the *Poll* frame from the *Initiator* it is issuing a *Response* frame with a defined delay of $\tau_{pr:tx}$. This *Response* includes time of reception $t_{p:rx}$ and time of transmission $t_{r:tx}$ on the *Responder* side. The *Initiator* is in turn enabling its receiver after $\tau_{pr:rx}$, setting a timeout of $\tau_{pr:to}$. Upon reception, the *Initiator*, knowing the reception time of the response $t_{r:rx}$ is then enabled to calculate the ToF $\tau_{tof:ss}$ as follows shown in Equation (1).

Since both clocks drift over the duration of this message, a clock drift compensation is conducted using a correction factor $\epsilon_{i,r}$ based on an estimate of the remote transmitter’s frequency offset. This is done on the firmware level by simply reading the correlation offset from the transceiver.

(1) $$\tau_{tof:ss}=\frac{\left(t_{r:rx}-t_{p:tx}\right)-\left(t_{r:tx}-t_{p:rx}\right)}{2}\;\cdot \;\left(1-\epsilon_{i,r}\right)$$

Due to the potential availability of the ranging result on both nodes and inherent clock drift compensation, the other popular ranging variant is the Double-Sided (DS) TWR. Here, after a duration of $\tau_{rf:tx}$ an additional *Final* frame is transmitted by the *Initiator* at $t_{f:tx}$ and received by the *Responder* at $t_{f:rx}$. In order to do this, the *Responder* is enabling its receiver after $\tau_{rf:rx}$ following the transmission of the *Response*, setting a *Final* reception timeout of $\tau_{rf:to}$. Therefore, for DS-TWR the ToF $\tau_{tof:ds}$ can be calculated as shown in Equation (2).

(2) $$\tau_{tof:ds}=\frac{\left(t_{r:rx}-t_{p:tx}\right)\;\cdot \;\left(t_{f:rx}-t_{r:tx}\right)-\left(t_{r:tx}-t_{p:rx}\right)\;\cdot \;\left(t_{f:tx}-t_{r:rx}\right)}{\left(t_{r:rx}-t_{p:tx}\right)+\left(t_{f:rx}-t_{r:tx}\right)+\left(t_{r:tx}-t_{p:rx}\right)+\left(t_{f:tx}-t_{r:rx}\right)}$$

Depending on the ranging setting the static node at which logging is performed changes as illustrated in Table 2. Logging is always performed at the static node. Due to the different availability of the final ranging results, the logging node is either the *Initiator* for SS-based configurations or the *Responder* for DS-based ranging.

The detailed timings for the ranging configurations used in the experiments are listed in Table 3. In order to achieve comparability, the goal was to keep the ranging frequency similar in all experiments. Since the main frequency is controlled through a simple waiting period $\tau_{d}$ between ranging attempts at the *Initiator* different values had to be chosen.

Going into that detail when considering or quantifying multi-user interference is important. The longer a receiver is active, the higher the probability for picking up interference. Therefore, the goal for resilient communication should be having very narrow timing margins in order to keep energy consumption at a minimum, but also to decrease the risk of picking up interference. Additionally, covering the different modes such as DS and SS in the legacy IEEE 802.15.4a variant DS-a and SS-a and the new IEEE 802.15.4z variant DS-z and SS-z the gives the informed system designer more information about the performance under interference. This might also help in the decision whether a move to new hardware or protocols involving breaking changes such as non-compatible STS modes is desirable, or using legacy equipment is still viable.

## 3. Experimental Evaluation

### 3.1. Dynamic Ranging Accuracy

**Figure 3:**
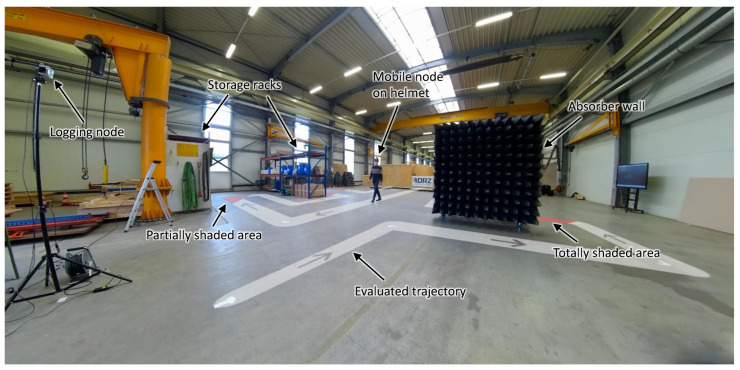
Illustrated photo of the scenario for dynamic ranging accuracy evaluation. A trajectory with a variety of absorption regions is evaluated under a large motion-capture installation for ground truth measurement.

**Figure 4:**
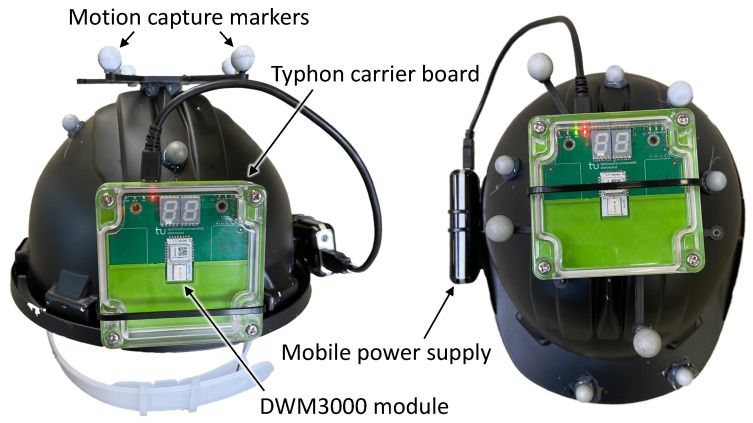
Pictures of the two helmet orientations used in the experiments. On the left hand side, the back-mounted tag is depicted. On the right hand side, the top-mounted tag is shown. Here, the tag is lying flat on the top of the helmet. Note that both configurations are equipped with motion capture markers for ground-truth generation.

In order to evaluate the ranging accuracy of different configurations for realistic scenarios, a multi-shading-region experiment in this context referred to as *experiment A* was conducted as illustrated in Figure 3. Here, the mobile unit was moved either on top or on the back of a motion capture-tracked helmet, see Figure 4 while the logging unit remained static. Both units were placed or carried at the same height of 2 m.

As illustrated, the tag is exposed to various degrees of shading, either artificial through an absorber-wall or natural shading for example through a storage rack. For the top-mounted case, there is no direct orientation dependent shadowing, where in the back-mounted case, the tag is shadowed through the head of the carrying person. The whole experimental area is covered by a large-scale *Qualisys* motion capture system with 21 *Miqus M3* cameras observing from the ceiling and 8 *Arqus A5* cameras tracking from the corners utilizing passive markers on the helmet to which the mobile node is mounted to. While the theoretical accuracy of this configuration is below 1 mm, we expect an accuracy in our measurements well below 1 cm.

**Figure 5:**
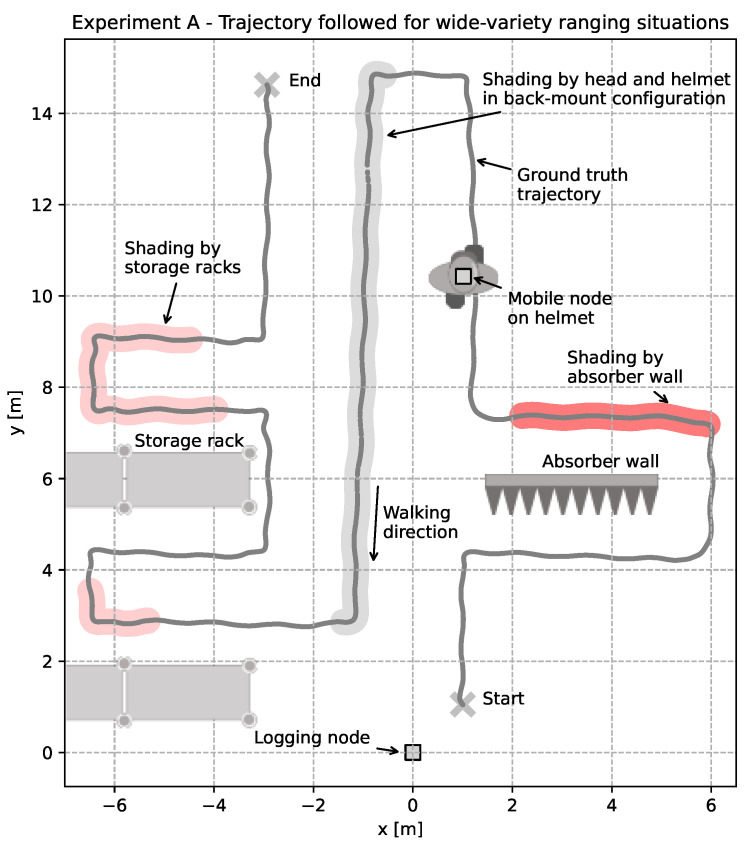
Schematic top-down illustration of the scenario for dynamic ranging accuracy evaluation. A trajectory through different regions of absorption and interference is followed under continuous tracking by a motion capture system.

A top-down view of the evaluation trajectory in *experiment A* is depicted in Figure 5. Due to artificial and natural features in this trajectory, a wide variety of ranging environments is covered by this experiment design. Artificial features such as an absorber wall enable the performance evaluation through a very effective non-line of sight condition. Natural features such as the storage racks in the evaluation environment provide partial shading.

Additionally to the shading without interference, extra nodes were distributed in the evaluation area to generate interfering traffic. This distributed interference is realized through three spatially distributed DWM3000 modules on carrier boards. The positions of the nodes were chosen around the walking trajectory at approximately x,y,z=[0,0,0.1],[0,17,1.2],[10,19,1.2]. The channel number is set as $n_{ch}=5$ and the preamble code to $c_{pr}=9$. Due to this, the generated frames are directly interfering with the evaluated rangings. It should be noted that a non-standard 8 symbol SFD was chosen such that preambles are interfering but successful SFD detection and decoding should not occur at the ranging pair. The interference frequency is limited by a waiting period of 10 ms. This results in an average interfering frame frequency of 100 Hz.

**Figure 6:**
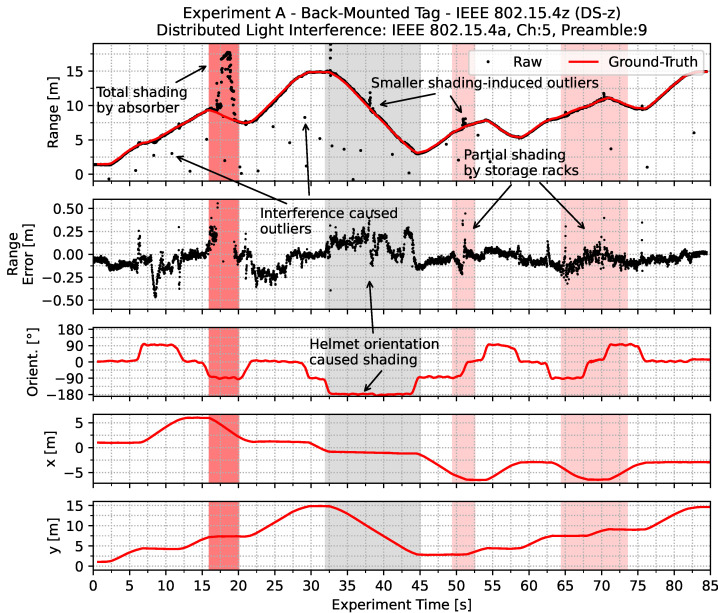
Timeseries of a single dynamic ranging accuracy evaluation trajectory. The different regions of obstruction become visible in the timeseries. With total shading by an absorber wall, rangings take an indirect path. With partial shading, noise increases but the rangings results remain mainly consistent. Note the interference-caused outliers throughout the whole experiment time.

An exemplary timeseries of double-sided two-way ranging with IEEE 802.15.4z based frames is depicted in Figure 6. The individual shading regions are highlighted consistently with the color scheme chosen in Figure 5. Next to the measured range, ground-truth values, Cartesian coordinates, orientation and the resulting ranging errors are shown in the timeseries. It is clearly visible that the different shading regions affect the resulting ranging results to different extends. Due to the total shading by the absorber, reception through indirect paths is caused, resulting in highly erroneous ranging results. The path the signal needs to take is higher than the path in line of sight would be. Therefore, a positive ranging error in the range of 5m to 10m is observed. Similar effects can be seen in the other shading regions, but with smaller periods, deflected paths and therefore, smaller ranging errors in the range of 1m–2m.

The effect of the distributed light interference is visible through negative outliers in the estimated ranges. This effect is due to an overlay of interfering frames in the channel and therefore, erroneous first path detection in the correlation of the reference pulse with the received signal at the receiving part of the ranging pair.

In order to evaluate the performance implications of using IEEE 802.15.4a in contrast to IEEE 802.15.4z based ranging pairs, a set of experiments is conducted within the experimental scenario of *experiment A*. Therefore, the experiment was repeated with DS and SS TWR with both standards in top- and back-mount configuration, with and without distributed light interference. Note that *DS-z* and *SS-z* based experiment runs were using samples of the *Qorvo DWM3000* and the *DS-a* and *SS-a* were using *Qorvo DWM1000 (former Decawave)* modules on the same carrier board, with the same case and mounting positions. Note that the different module positions depicted in Figure 4 are accounted for in the ground-truth measurements by an orientation dependent transformation. All experiments were conducted sequentially and the raw experimental data is provided alongside this work.

**Figure 7:**
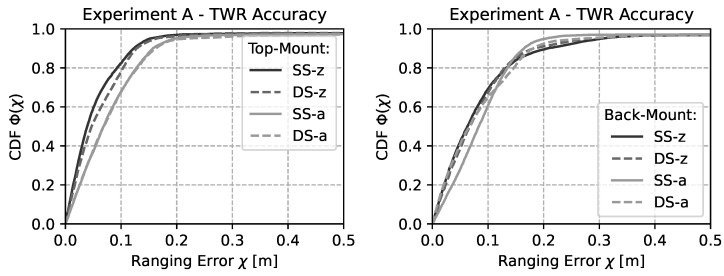
CDF of the ranging accuracy during the experiment for different ranging schemes without active interference. The performance of DS and SS TWR is similar for both the IEEE 802.15.4a and the IEEE 802.15.4z based rangings.

A Cumulative Distribution Function (CDF) of the experimental results for the top- and back-mount configuration without active interference are depicted in Figure 7. It is clearly visible that both single-sided and double-sided ranging delivers a similar accuracy. Further, there is no large difference between the IEEE 802.15.4a and the IEEE 802.15.4z based range results. However, there is a relatively small difference between top- and back-mounted mobile nodes.

**Figure 8:**
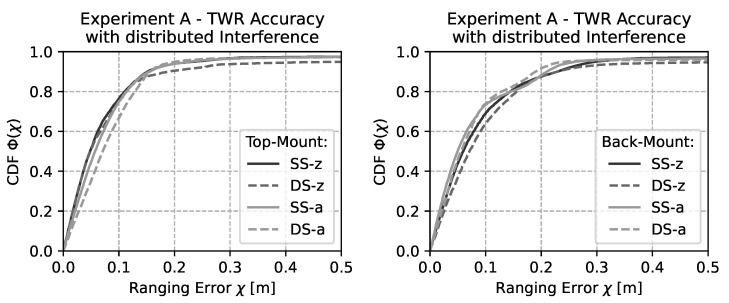
CDF of the ranging accuracy with spatially distributed interference. The overall performance is influenced by sparse but relatively large outliers that mainly affect DS-z.

In the next set of experimental runs light active distributed interference is added to the environment. The resulting CDF is depicted in Figure 8. It is clearly visible that due to the light interference the overall performance is similar to the performance without interference. Therefore, it can be stated that the observed ranging performance (setting aside interference) will be quite comparable between the legacy IEEE 802.15.4a UWB PHY and the new IEEE 802.15.4z UWB PHY. However, the large range outliers that are also visible in Figure 6 are introducing severe errors in around 1% of the cases for *DS*-z as were previously observed in Figure 6.

While these errors appear to be sparse having only a negligible influence to the overall ranging performance, they can be a disabler for many applications. Any application with strong requirements in robustness and reliability suffers from these uncertainties. With wider usage of UWB transmissions such as the integration in current phones and accessories as well as industry deployment, the interference level that is generally expected increases. Therefore, two other experiments are conducted to quantify these effects and show potential mitigation approaches by the new IEEE 802.15.4z standard and manufacturers.

### 3.2. Controlled Interference

**Figure 9:**
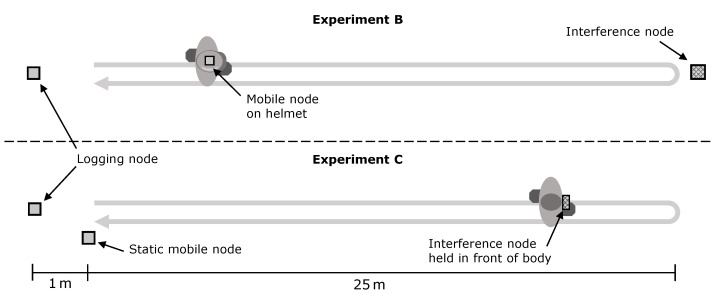
Schematic illustration of the scenario for controlled interference evaluation. Two experiments were conducted with controlled interference. In experiment *B* the interferer is static and not directly obstructed. In experiment *C* the interferer is mobile and carried in front of the body, obstructing the LOS to the ranging pair in the first half of the experiment. In the second half, the interferer is facing towards the ranging pair and has therefore, direct LOS while coming closer to both.

To investigate the effect of interference on the ranging results using in a quantifiable manner with different configurations, two experiments were conducted under controlled interference as shown in Figure 9. In both experiments, a straight line of 25 m is walked forth and back. In *experiment B*, ranging is performed between the helmet-mounted mobile node and the static logging node. The interference node is statically located at the end of the walked path at 25 m distance to the logging node. Whereas in *experiment C* the interference node is carried along the path, being held in front of the body. Throughout the experiment, the logging node attempts ranging with the mobile node, which is statically placed at a distance of 1.20 m. All units were placed or carried at the same height of 2 m.

For the controlled interference, a single DWM1000 module on our carrier board was chosen. Here, the channel number $n_{ch}$ and the preamble code $c_{pr}$ were varied using the settings listed in Table 1. In order to evaluate interfering IEEE 802.15.4a-2011 traffic, no STS was used. Further, a standard SFD was used. For the interference in $n_{ch}=9$, a DWM3000 module with the same settings, but a non-standard 8 symbol SFD was used.

The interference frequency is set to a maximum, such that the interferer node is transmitting frames without waiting periods. This results in an average interfering frame frequency of around 4.3kHz–4.5kHz at an average inter-arrival time of 220 μs–230 μs.

Please note that as with *experiment A*, *experiment B* and also the upcoming *experiment C* are actual experiments with actual transceivers in a real physical environment.

In order to illustrate the effect of the interference, a set of seven different ranging configurations was evaluated in *experiment B*, with the basic parameters listed in Table 2 and Table 3. Each ranging configuration was repeated with and without the interference through a static interferer transmitting at a high rate using IEEE 802.15.4a compatible blink frames on the same channel and preamble code as the ranging pair, namely channel 5 with preamble code 9. Further, it should be noted that for this experiment the mobile node is mounted in *Top* configuration as illustrated in Figure 4.

**Figure 10:**
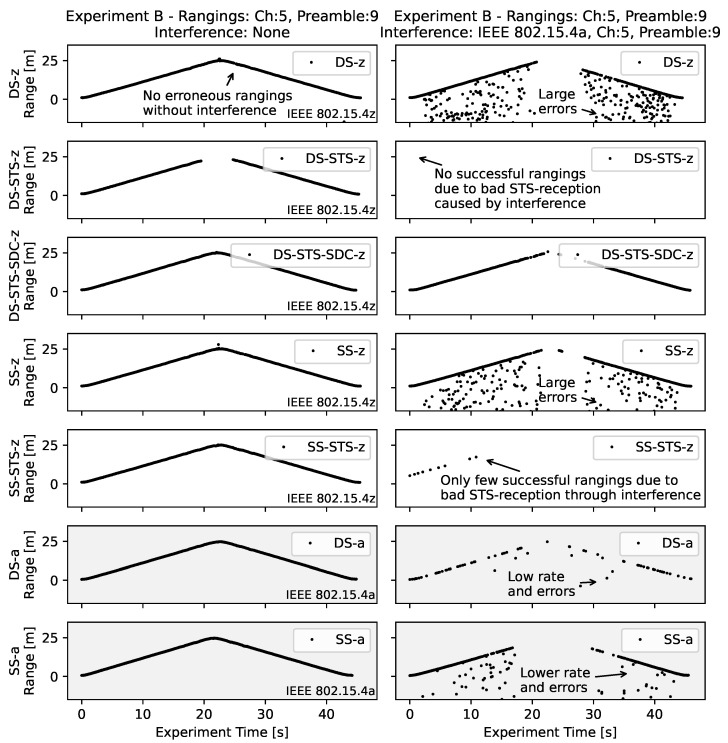
Timeseries of the controlled interference evaluation with a mobile ranging node for a variety of ranging schemes and configurations in *experiment B*. On the left hand side the experiment without interference is depicted. On the right hand side IEEE 802.15.4a based interference is introduced. Note that the effect of this active in-channel interference is significantly influencing the ranging results. While with basic ranging without STS large errors are introduced, STS is preventing this effect at the cost of few to no successful rangings.

The resulting timeseries of the 14 experiment runs are depicted in Figure 10. On the left hand side the ranging results (estimated distance) without active interference is shown. The walking trajectory shows clearly in the triangular form of the rangings. It is also clearly visible that there are few to no outliers and gaps in the resulting graphs. On the right hand side, however, the interference-induced effects vary greatly with the ranging configuration. Large negative errors in the estimated range can be observed for the basic single-sided and double sided ranging schemes without STS. Further, lower rates and partial outages in the area in which the mobile node is getting closer to the interferer can be observed. Especially the IEEE 802.15.4a based double-sided ranging *DS-a* is prone to the kind of interference introduced in this experiment.

Observing the STS-based ranging schemes *DS-STS-z* and *SS-STS-z*, there is almost no successful ranging possible within the duration of the experiment. Here, the interference is preventing successful reception such that the receiver is rejecting frames due to inconsistencies. In this environment, the only gracefully degrading configuration is *DS-STS-SDC-z*. Though there is degradation in rate, largely erroneous rangings are not observed in this experiment.

The effects depicted in Figure 10 illustrate the shortcomings of basic IEEE 802.15.4a and IEEE 802.15.4z based TOA estimation without cryptographic elements in the frame structure itself. The large interference-induced errors can be highly problematic for many applications that expect certain error distributions and consistency of the rangings. For practical applications, these occasional outliers are very hard to detect and pose a big issue energy-constrained nodes, where potential mitigation by frequent ranging repetitions come at a very high cost in battery life, making certain applications unfeasible.

Moreover, depending on the quality of the channel, ranging configuration and protocol-induced effects can lead to gaps in coverage-edge scenarios such as observable in Figure 10 in the DS-STS-z timeseries without interference.

In order to quantify the observed effects for comparison *experiment C* is conducted. Here, the signal to interference ratio is actively influenced through interferer mobility and shadowing while the ranging pair remains static. The same set of ranging configurations as used in *experiment B* was used in this experiment. However, interference now used IEEE 802.15.4z based blink frames, but with the same channel and preamble code as used for the ranging pair. Please note that throughout the experiment, the interferer was held in front of the chest of the carrying person. Therefore, it was shaded through the body in the first half of the experiment while the person is walking away from the ranging pair, resulting in a NLOS condition. In the second half the interferer is facing towards the ranging pair with LOS increasing the effect of the interference.

**Figure 11:**
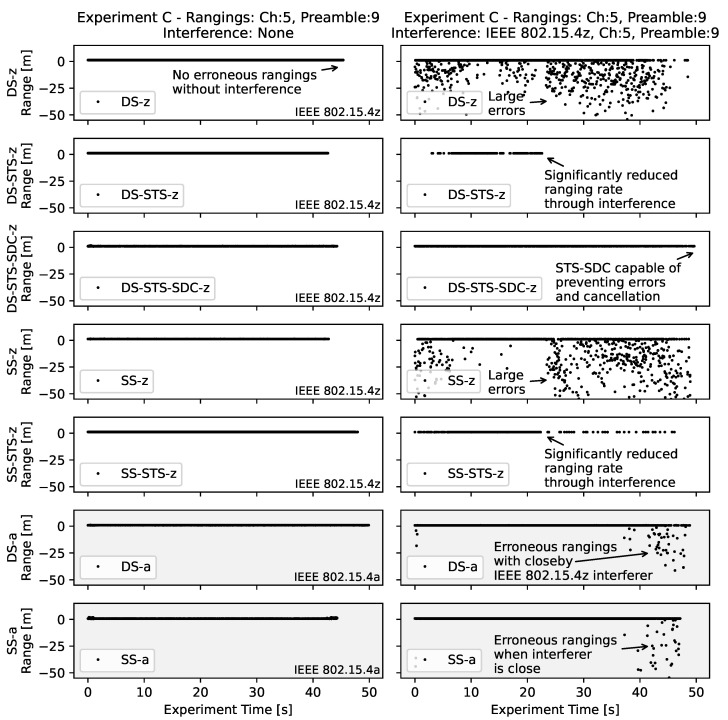
Timeseries of the controlled interference experiment *C* for the static ranging pair. On the left hand side, no active interference is introduced. On the right hand side active IEEE 802.15.4z interference is introduced. The interferer is carried first away from the ranging pair in NLOS, then back facing towards the ranging pair with LOS. Note the different effects of the interference for different ranging schemes and configurations.

The resulting fourteen timeseries are depicted in Figure 11. On the left hand side the ranging estimates without interference are shown. It is clearly visible that neither rate nor accuracy varies much for these experiments.

On the right hand side however, with active interference large negative errors are introduced in the rangings for the single- and double-sided ranging configurations without cryptographic additions such as the STS. In contrast to *experiment C* more successful rangings are observed generally. This is due to the increased signal to interference ratio. The ranging pair is close to each other at about 1.2 m and the interferer is moved from both in contrast to between the initiator and the responder. The point at which the interferer-carrying person turns towards the ranging pair can be seen through a sudden increase in erroneous rangings.

It should be noted that due to the IEEE 802.15.4z based Interference, the rangings that are based on the old IEEE 802.15.4a standard *DS-a* and *SS-a* suffer only from erroneous range results when the interferer is close by. When observing the STS-based ranging configurations it can be clearly seen that there are no erroneous rangings but the rate reduces significantly once the interferer is in LOS to the ranging pair. Similar to *experiment B*, the SDC-based approach is capable of mitigating erroneous range results, but also maintaining a usable rate.

**Figure 12:**
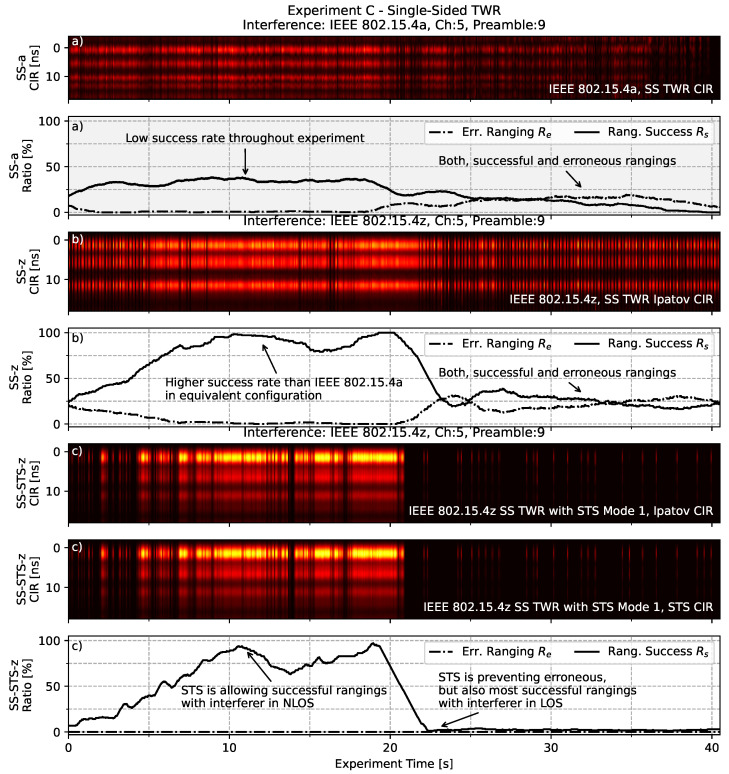
Illustrative timeseries of three runs of the controlled interference *experiment C*. Next to a floating success- and error rate for the rangings the accumulator based CIR is depicted. Note the difference in success- and error rates under interference for plain IEEE 802.15.4a in subfigure (**a**) and plain IEEE 802.15.4z SS TWR in subfigure (**b**). Also note the difference to the STS enhanced variant employed by IEEE 802.15.4z depicted in subfigure (**c**).

In order to illustrate this effect further, Figure 12 shows an exemplary comparative timeseries of single-sided TWR with interference in the context of *experiment C*. Here, a timeseries of the Channel Impulse Response (CIR) captured in the accumulator of the logging node is plotted next to a moving average of the ranging success rates $R_{s}=\left(N_{s}-N_{e}\right)/N$ and the erroneous range rates $R_{e}=N_{e}/N$. For visual improvement the timeseries of the success- and outlier-ratios are moving average filtered observing a time-span of 3 s. Here, *N* is the total number of expected rangings in the observed time-span, calculated based on the average ranging interval. $N_{s}$ is the raw number of successfully received rangings as recorded in the observed time-span. $N_{e}$ is the number of erroneous rangings in the observed time-span at which the reported range differs more than 0.2 m from the measured distance.

The upper section of Figure 12a shows the *SS-a* ranging based on the IEEE 802.15.4a standard under interference in the same standard, channel and preamble code. It is clearly visible that the success rate of frames is relatively low with an average of around 35% while the interferer is in NLOS and moving away from the ranging pair. The rate of erroneous range results is comparably low in this first half of the experiment. However, once the interferer is in LOS and turned towards the ranging pair, the rate of successful rangings drops and the rate of erroneous range results increases significantly to around 20%. Please note the spots in the heatmap above 0 ns. Here, the correlation with the reference pulse is strong before the first path is even detected, indicating potentially erroneous ranging results.

The middle part of Figure 12b shows the result for the *SS-z* ranging based on the IEEE 802.15.4z standard. It is clearly visible that there is an improvement in terms of a higher success rate than the IEEE 802.15.4a ranging in equivalent configuration. When the interferer moves sufficiently from the ranging pair, the ranging success rate averages at around 85%. However, once turned towards the ranging pair creating an LOS condition, the success rate drops and the rate of erroneous ranging results increases to around 25%. In this constellation about half of the conducted rangings deliver erroneous range estimations.

The lower section of Figure 12c on the other hand shows the effect of STS with *SS-STS-z* in the same experimental setup. While being able to successfully range in the NLOS section of the experiment, the successful ranging rate drops to 2% when the interferer is in LOS to the ranging pair. However, this prevents erroneous rangings as observed with the other ranging schemes.

**Figure 13:**
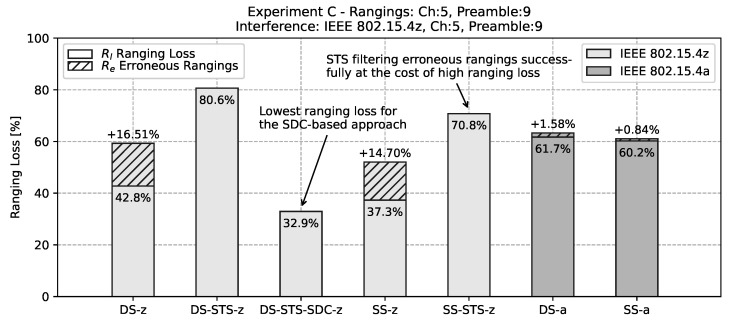
Bar chart of the ranging loss (the percentage of trials that did not lead to a ranging result) for experiment *C*. Next to the unsuccessful rangings the percentage of trails that led to erroneous rangings with errors greater 0.2 m is shown. Note the strong cancellation effect of STS in contrast to plain TWR.

In order to quantify the results of *experiment C* a bar chart of the ranging loss $R_{l}=1-\left(N_{s}/N\right)$ and the total erroneous rangings over the full experiment duration is depicted in Figure 13. While the absolute numbers are the result of this specific interference scenario with controlled interference, the observed effects are expected to correlate with the relative ranging scheme performance in practical conditions.

In Figure 13 it is clearly visible that as observed qualitatively in Figure 11, the ranging schemes without cryptographic components *DS-z* and *SS-z* have a large percentage of range results with erroneous readings. Similarly the IEEE 802.15.4a-based ranging schemes *DS-a* and *SS-a* have high ranging loss rates but smaller erroneous readings. As observed in the timeseries, the STS-based approaches *DS-STS-z* and *SS-STS-z* is filtering erroneous rangings successfully on the cost of a high ranging loss. It is clearly visible that with in-band and same preamble code interference the SDC based approach *DS-STS-SDC-z* offers a good trade-off between actual achievable ranging rate while preventing large erroneous rangings.

For practical setups where multiple UWB localization systems are in place it is close at hand to operate systems in parallel using different preamble codes or channels to increase signal to interference ratio. Due to the independent nature of these scenarios, systems and transmissions are likely to be uncoordinated. Therefore, a set of three additional experiments is performed.

**Figure 14:**
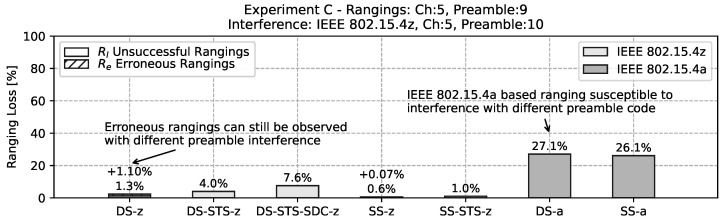
Bar chart of the ranging loss for interference with non-matching preamble codes but within the same channel. Note that with a ranging loss of over 25% the IEEE 802.15.4a based ranging is still strongly influenced by the interferer.

In the first experiment, based on the mobility of *experiment C* the same channel, but a different preamble code is chosen. The results are depicted in the bar chart in Figure 14. Here, the basic TWR schemes without cryptographic elements *DS-z* and *SS-z* have low to no ranging loss, but still a small percentage of erroneous range readings. The IEEE 802.15.4a based ranging schemes *DS-a* and *SS-a* is still susceptible to this kind of interference, with a ranging loss of around 25%. When adding STS double-sided ranging *DS-STS-z* the erroneous rangings are still successfully filtered but at the cost of a ranging loss of around 4%. In this context the single-sided variant is not susceptible to this kind of interference. Further, the addition of SDC is introducing a relatively large ranging loss. This might be due to the different PHY configurations as mentioned in Section 2.

**Figure 15:**
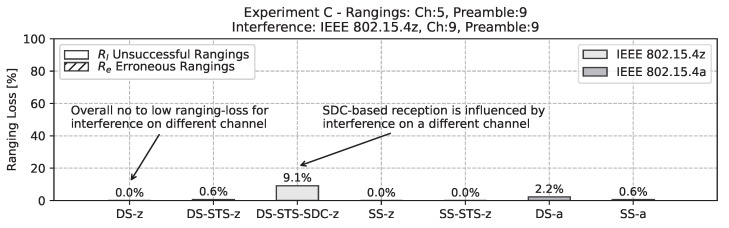
Bar chart of ranging loss for interference with non-matching channels while using the same preamble code. Note the absence of erroneous ranging results. Moreover, the SDC-based approach is influenced notably by the interference.

The second additional experiment in the mobility scenario of *experiment C* is using a different channel for the interference, but the same preamble code. Here, the interference is in channel 9 at with a center frequency of around 8 GHz while the ranging pair remains at channel 5 with a center frequency of around 6.5 GHz. As depicted in Figure 15 most schemes are barely influenced by the out-of-channel interference. However, the SDC-based approach has a slightly increased ranging loss in this scenario at around 9%.

**Figure 16:**
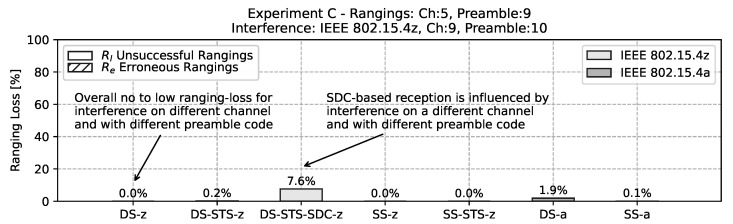
Bar chart of ranging loss for interference with non-matching channels and non-matching preamble codes. Note the overall low effect on most approaches except for the SDC-based method.

In the third additional experiment utilizing the mobility scenario of *experiment C* a different channel and a different preamble code is used for the interferer. With an IEEE 802.15.4z based interferer at channel 9 and preamble code 10 a similar picture shows. As depicted in the bar chart in Figure 16 most ranging schemes are not affected by the interference in this scenario. However, the SDC based ranging *DS-STS-SDC-z* again experiences a relatively large ranging loss under interference of around 7%. While this is counter-intuitive, it is assumed that the manufacturer defaults for the SDC are not handling the interference with different preamble lengths and SFD configuration well. It could be that with overall consistent configuration (which can be achieved in controlled environments) the performance of SDC is better than plain STS under interference.

In general it can be stated that if reliable ranging or TOA estimation is required for the usage scenarios, the utilization of STS is a must. The results presented in this section could that the integrity of the rangings with all other approaches cannot to be guaranteed under malicious interference. Therefore, DS-STS-z and SS-STS-z are the only settings that should be used if these requirements need to be met. Further, for ranging or localization with no critical dependency on the security side such as a trusted environment with legacy transceivers, the SDC addition might improve throughput on the cost of being susceptible to targeted attacks. If there is no critical dependency on TOA integrity, such as plain encrypted data transmission, the STS component might lead to additional packet loss. Therefore, for more complex systems hybrid strategies such as using STS for TOA estimation and omitting it for data transfer might be beneficial.

## 4. Conclusions and Future Work

This work provides an experimental evaluation of the new IEEE 802.15.4z UWB PHY under multi-user interference. The evaluation features a set of three different experiments in order to assess the qualitative and quantitative effects on the ranging capabilities in different configurations. The first experiment showed the practical effects on the ranging results in a typical scenario with different LOS and NLOS situations with or without interference. We showed that outliers can be provoked even with spatially distributed low frequency UWB interference. The second experiment illustrated these effects with controlled interference, severely limiting the performance depending on the chosen configuration. In the third experiment a comparative quantification of the ranging throughput and erroneous ranging rate could be provided, depending on the configuration choices for the UWB PHY.

Overall it could be shown that novel additions to the standard, such as the STS are mostly capable of preventing erroneous ranging results, at the cost of a significantly reduced ranging rate in the presence of multi-user interference. If safety or security related applications or usage scenarios are envisioned with UWB based communication and localization, the utilization of STS is a must. We could show that all other approaches are highly susceptible to malicious interference. Other approaches proposed by some manufacturers such as SDC may help countering this effect, but suffer from the lack of security and may introduce ranging loss under out-of-band interference with non-matching PHY config.

In future work it is planned to investigate the performance of different manufacturer transceivers as well as heterogeneous protocol implementations. Since most of the upcoming UWB solutions will be multi-communication-technology based, the resulting interoperability and multi-carrier interference will play a much larger role when device density increases. Additionally, the effect of different PHY configurations is of strong interest due to the high heterogeneity in the market. An analysis with an additional degree of freedom such as preamble length may lead to interesting results.

## Figures and Tables

**Table 1:** Basic common channel configuration. Note that in case of STS usage, $n_{sts}$ symbols are used. Further, the corresponding standard PHR and SFD are used throughout the experiments.

Chan.	Preamble	PRF [MHz]	*R* [Mbps]	$c_{\mathrm{pr}}$	PAC	$n_{\mathrm{sts}}$
5	128	62.4	6.8	9	8	128

**Table 2:** Basic parameters of the settings for two-way ranging used in the experiments.

Setting	Standard	Rng. Type	Logging	STS
DS-z	IEEE 802.15.4z	Double-Sided	Responder	-
DS-STS-z	IEEE 802.15.4z	Double-Sided	Responder	Mode 1
DS-STS-SDC-z	IEEE 802.15.4z	Double-Sided	Responder	Mode 1 + SDC
SS-z	IEEE 802.15.4z	Single-Sided	Initiator	-
SS-STS-z	IEEE 802.15.4z	Single-Sided	Initiator	Mode 1
DS-a	IEEE 802.15.4a	Double-Sided	Responder	-
SS-a	IEEE 802.15.4a	Single-Sided	Initiator	-

**Table 3:** Timing parameters for the two-way ranging used in the experiments.

Setting	$\tau_{\mathrm{pr}:\mathrm{tx}}$	$\tau_{\mathrm{pr}:\mathrm{rx}}$	$\tau_{\mathrm{pr}:\mathrm{to}}$	$\tau_{\mathrm{rf}:\mathrm{tx}}$	$\tau_{\mathrm{rf}:\mathrm{rx}}$	$\tau_{\mathrm{rf}:\mathrm{to}}$	$\tau_{d}$
DS-z	900 μs	700 μs	300 μs	700 μs	500 μs	220 μs	15 ms
DS-STS-z	900 μs	690 μs	300 μs	880 μs	500 μs	220 μs	15 ms
DS-STS-SDC-z	900 μs	690 μs	300 μs	880 μs	670 μs	300 μs	15 ms
SS-z	450 μs	240 μs	210 μs	-	-	-	17 ms
SS-STS-z	950 μs	700 μs	700 μs	-	-	-	17 ms
DS-a	900 μs	700 μs	300 μs	700 μs	500 μs	220 μs	15 ms
SS-a	450 μs	240 μs	510 μs	-	-	-	10 ms

## Sample Availability

Samples of the compounds [37] are available from the authors.

## Abbreviations

The following abbreviations are used in this manuscript:

CDF	Cumulative Distribution Function
CIR	Channel Impulse Response
DS	Double-Sided
IEEE	Institute of Electrical and Electronics Engineers
NLOS	Non-Line of Sight
LOS	Line of Sight
PAC	Preamble Acquisition Chunk
PHR	Packet Header
PHY	Physical Layer
PRF	Pulse Repetition Frequency
SDC	Super Deterministic Code
SFD	Start of Frame Delimiter
SS	Single-sided
STS	Scrambled Time Sequence
SYNC	Synchronization Preamble
TDMA	Time-Division Multiple Access
TDOA	Time-Difference of Arrival
TOA	Time of Arrival
TOF	Time of Flight
TWR	Two-Way Ranging
UWB	Ultra-Wideband
